# Optimization and Stability Assessment of *Monochamus alternatus* Antimicrobial Peptide MaltAtt-1 in *Komagataella phaffii* GS115 for the Control of Pine Wood Nematode

**DOI:** 10.3390/ijms25168555

**Published:** 2024-08-06

**Authors:** Di Jiang, Xuhuizi Xu, Zeguang Wang, Chao Yu, Zeqing Wang, Yuda Xu, Xu Chu, Ming Li, Feiping Zhang, Xia Hu

**Affiliations:** 1 Forestry College, Fujian Agriculture and Forestry University, Fuzhou 350002, China; 2 Key Laboratory of Integrated Pest Management in Ecological Forests, Fujian Agriculture and Forestry University, Fuzhou 350002, China

**Keywords:** antimicrobial peptide, induction optimization, *Komagataella phaffii*, stability

## Abstract

MaltAtt-1 is an antimicrobial peptide isolated from *Monochamus alternatus* with nematocidal activity against pine wood nematode. In this study, a eukaryotic expression system based on *Komagataella phaffii* GS115 was established, and its secretory expression of MaltAtt-1 was realized. The basic properties and secondary and tertiary structures of the antimicrobial peptide MaltAtt-1 were identified by bioinformatics analysis. MaltAtt-1 is a hydrophilic stable protein, mainly composed of an α-helix (Hh), β-folds (Ee), and irregular curls (Cc). The optimal fermentation conditions for MaltAtt-1 were determined by a single-factor test and the Box–Behnken response surface method, including an induction time of 72 h, induction temperature of 30 °C, culture medium of pH 7.6, methanol volume fraction of 2.0%, and an initial glycerol concentration of 1%. The stability of MaltAtt-1 indicated its resistant to UV irradiation and repeated freezing and thawing, but the antibacterial activity decreased significantly under the influence of high temperature and a strong acid and base, and it decreased significantly to 1.1 cm and 0.83 cm at pH 2.0 and pH 10.0, respectively. The corrected mortality of *B*. *xylophilus* achieved 71.94% in 3 h at a concentration of 300 mg·L^−1^ MaltAtt-1 exposure. The results provide a theoretical basis for the antimicrobial peptide MaltAtt-1 to become a new green and efficient nematicide.

## 1. Introduction

Pine wilt disease (PWD) is a systemic disease with a wide host range, rapid spreading speed, and high mortality rate. It is caused by the pine wood nematode, *Bursaphelenchus xylophilus* (PWN) [1,2,3]. PWD control is an urgent topic, and, at present, it is mainly carried out based on a chemical control method using synthetic nematicides [4,5,6]. However, large-scale application of these synthetic chemicals has brought various concerns regarding forest ecosystems, as it may result in environmental pollution, toxicity to non-target organisms, and drug resistance [7,8], making it necessary to develop more effective and eco-friendly methods to prevent PWN infection. Biological control relying on eco-friendly agents, mainly derived from plant and microorganism resources, is a promising method [9,10].

Antimicrobial peptides (AMPs) are widely distributed host defense molecules that are produced by all multi-cellular plants and animals as well as by certain single-cell organisms, including prokaryotic and eukaryotic organisms [11,12,13]. Antimicrobial peptides have a broad spectrum of antimicrobial activity and are less likely to induce drug resistance in target bacteria or fungi [14,15]; furthermore, research has shown that AMPs may also be fatal to nematodes, whether they are gastrointestinal parasites or phytoparasites [16,17]. In our previous study, we identified a novel attacin AMP, MaltAtt-1, from *Monochamus alternatus*. The MaltAtt-1 expressed by *Komagataella phaffii* KM71 exerted a potent antimicrobial activity against multiple species of bacteria and, moreover, had a certain nematocidal effect on *B*. *xylophilus*: at a concentration of 0.19 mg·mL^−1^, it achieved half death on *B*. *xylophilus* in 3 h, and the toxicity activity was 98.7% at 0.3 mg·mL^−1^ after 6 h of treatment, making it a potential new-generation eco-friendly nematicide [18].

The *K. phaffii* expression system has been used extensively for heterologous protein production, primarily due to the ease with which it can be propagated and its inherent ability to secrete large amounts of protein [19]. In previous studies, KM71 was selected as the host strain for expression of the recombinant protein MaltAtt-1, and the expression system of *K*. *phaffii* was successfully established as a result [18]. However, the expression mode of the recombinant protein by KM71 was intracellular, which made the process of obtaining the protein complicated. In fact, *K*. *phaffii* is a suitable microorganism for the secretory production of recombinant proteins directly into the supernatant of the culture medium in the case of selecting a suitable strain [20]. The limited production mode of endogenous secretory proteins by the *K*. *phaffii* expression system makes purification of the recombinant protein easy; at the same time, high levels of production, especially in the case of unstable peptides, necessitate optimization to some extent. Conventional culture condition optimization (regarding pH or methanol concentration) may also increase the production of heterologous peptides in *K*. *phaffii*. It has been reported that the yield of recombinant protein produced in *K*. *phaffii* could be increased by more than 10-fold by optimizing the culture conditions [21,22].

In this study, the recombinant plasmid pPIC9K-MaltAtt-1 was transformed into *K*. *phaffii* GS115, enabling secretory expression. The basic properties and the secondary and tertiary structures of the antimicrobial peptide MaltAtt-1 were identified through bioinformatics analysis. Optimal conditions for the synthesis and secretion of MaltAtt-1 were determined using a response surface test, and the stability of MaltAtt-1 was assessed for further applications. The diameter of the inhibition zone in the fermentation supernatant served as an indicator of MaltAtt-1 production and antimicrobial efficacy. Additionally, the nematocidal activity of MaltAtt-1 produced via this novel secretion system was assessed for its effectiveness against *B*. *xylophilus*.

## 2. Results

### 2.1. Sodium Dodecyl Sulfate-Polyacrylamide Gel Electrophoresis (SDS-PAGE) of MaltAtt-1

**Figure 1:**
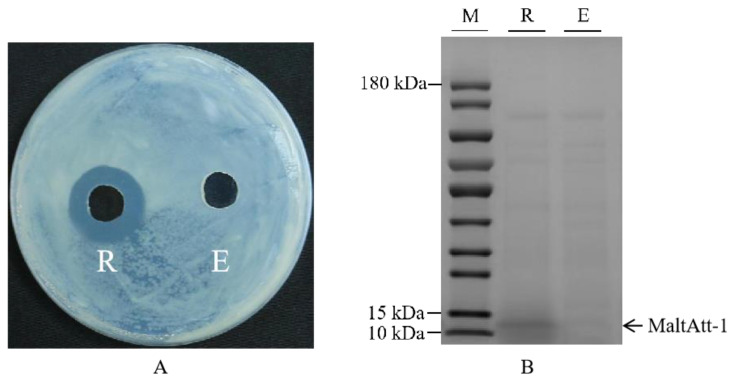
Inhibition zone and SDS-PAGE image of fermentation supernatant of *K*. *phaffii* GS115: (**A**) inhibition zone of fermentation supernatant of *K*. *phaffii* GS115; (**B**) SDS-PAGE image of fermentation supernatant of *K*. *phaffii* GS115. R: supernatant of *K*. *phaffii* GS115 with recombinant vector; E: supernatant of *K*. *phaffii* GS115 with empty vector; M: marker. *K*. *phaffii* GS115 strains were cultured in 30 °C, pH 7.0, with 2.0% methanol addition and 1.0% glycerol concentration.

Recombinant plasmid pPIC9K-MaltAtt-1 was chemically transformed into the *K*. *phaffii* GS115 strain, and induced expression was achieved using methanol as the only carbon source. The inhibition zone experiment showed that the fermentation supernatant of *K*. *phaffii* GS115 with a MaltAtt-1 recombinant vector had a significant antimicrobial activity compared to those with an empty vector (Figure 1A). SDS-PAGE identification and analysis were performed on the fermentation supernatant, proving that MaltAtt-1 could be secreted into the medium through induced expression (Figure 1B).

### 2.2. Bioinformatics Analysis of Antimicrobial Peptide MaltAtt-1

**Figure 2:**
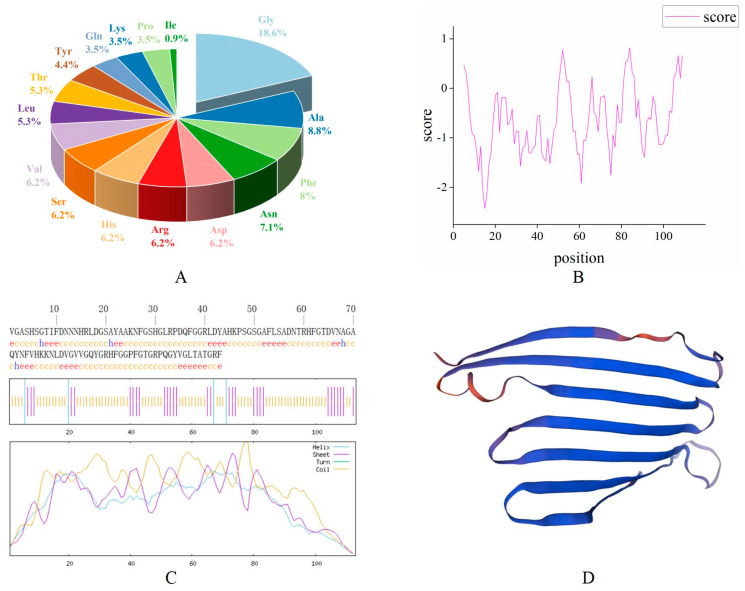
Bioinformatics analysis of the antimicrobial peptide MaltAtt-1 protein: (**A**) amino acid composition of MaltAtt-1; (**B**) hydrophilic/hydrophobic analysis of MaltAtt-1; (**C**) secondary structure prediction of MaltAtt-1; (**D**) tertiary structure prediction of MaltAtt-1.

The bioinformatics prediction analysis results showed that the antimicrobial peptide MaltAtt-1 is composed of 113 amino acid residues, with the largest proportion of glycine (Gly) (18.6%), followed by alanine (Ala) (8.8%) (Figure 2A). The molecular formula is C_528_H_782_N_164_O_158_, the relative molecular weight of the protein is 11,955.02, the theoretical isoelectric point is 9.69, the instability coefficient is 13.68, which is less than 40, proving that it is a stable protein, and the aliphatic coefficient is 50.97, reflecting the thermal stability of the protein and peptide. A higher aliphatic coefficient indicates greater protein stability (Table 1). Hydrophobicity analysis showed that the protein’s hydrophilic region has more hydrophobic regions, making it a hydrophilic protein (Figure 2B).

SOPMA was used to predict the secondary structure of the MaltAtt-1 protein. The results showed that the content of the α-helix (Hh) accounted for 3.54%, 31 β-folds (Ee) accounted for 27.43%, although there were no β-corners (Tt), and that 78 irregular curls (Cc) accounted for 69.03% of the secondary structure (Figure 2C). The results of the tertiary structure prediction were consistent with those of secondary structure prediction (Figure 2D).

### 2.3. Optimization of Induction Conditions for Recombinant Protein MaltAtt-1

**Figure 3:**
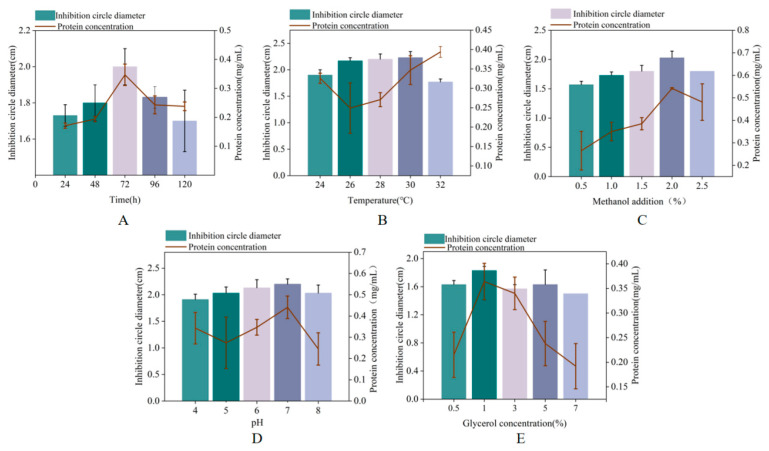
Effects of different induction conditions on MaltAtt-1 expression: (**A**) effects of different induction times on MaltAtt-1 expression; (**B**) effects of different induction temperatures on MaltAtt-1 expression; (**C**) effects of different methanol additions on MaltAtt-1 expression; (**D**) effects of different medium pH on MaltAtt-1 expression; and (**E**) effects of different initial glycerol concentrations on MaltAtt-1 expression.

We investigated the effects of induction time, temperature, pH, methanol concentration, and initial glycerol concentration on the production of MaltAtt-1. Antibacterial activity and MaltAtt-1 expression were evaluated by measuring the concentration and inhibition zone diameter against *Escherichia coli*. The antibacterial activity and expression of MaltAtt-1 increased initially with longer induction times, peaking at 72 h (Figure 3A). Considering time constraints and potential peptide degradation, subsequent single-factor tests were conducted with a 72 h induction period. During shake flask fermentation, the protein concentration generally increased between 26–30 °C, with the lowest antibacterial activity observed at 32 °C (Figure 3B). To preserve antimicrobial peptide activity, 30 °C was identified as the optimal induction temperature.

Methanol concentration significantly impacted cell growth and protein expression; optimal antibacterial activity and protein concentration were observed at 2% methanol, decreasing with concentrations exceeding 2.5% (Figure 3C). Excess methanol can be toxic to cells, adversely affecting protein expression. Medium pH during induction affected both the quantity and quality of foreign protein expression. Figure 3D shows that pH 8.0 resulted in the largest antibacterial zone diameter, indicating maximum antibacterial substance production and activity. Deviations from pH 8.0 led to reduced antibacterial activity, likely due to effects on the membrane charge and enzyme activity.

### 2.4. Model Analysis and Validation

Based on the results of the single-factor experiments, an orthogonal experiment was designed (Table S1), followed by the use of a response surface model to explore the interactions between these factors (Table 2). In the tables, the letters A, B, and C denote the independent effects of three single factors: induction temperature, medium pH, and methanol addition, respectively. Combinations, like AB, AC, and BC, represent the interactions between corresponding factors, while terms, like A^2^, B^2^, and C^2^, denote the quadratic effects of these factors. The significance values in the table indicate whether the model aligns with the actual experimental results. The model was found to be extremely significant (*p* = 0.0002), indicating the high feasibility of the quadratic regression equation used in this experiment. Conversely, the non-significant mismatch item (*p* = 0.3601 > 0.05) suggests that this aspect did not significantly affect the model’s validity. Furthermore, the determination coefficient (R^2^) of the experimental model was 0.9677, indicating a strong fit between the actual and predicted results of the antibacterial zone diameter. The adjusted determination coefficient (R^2^_Adj_ = 0.9263) demonstrates that the model effectively captures the variability in the response variable. This comprehensive analysis underscores the reliability and robustness of the experimental design and the predictive model used in this study.

A, C, AC, A^2^, B^2^, and C^2^ were significant terms of the model (*p* ˂ 0.05), indicating that the linear and square terms of induction temperature, the linear and square terms of medium pH, the linear and square terms of methanol addition, and the term of interaction between temperature and methanol addition were significant (Table 2). The other terms, including B, AB, and BC, were not significant in terms of the expression of the recombinant protein (*p* > 0.05).

**Figure 4:**
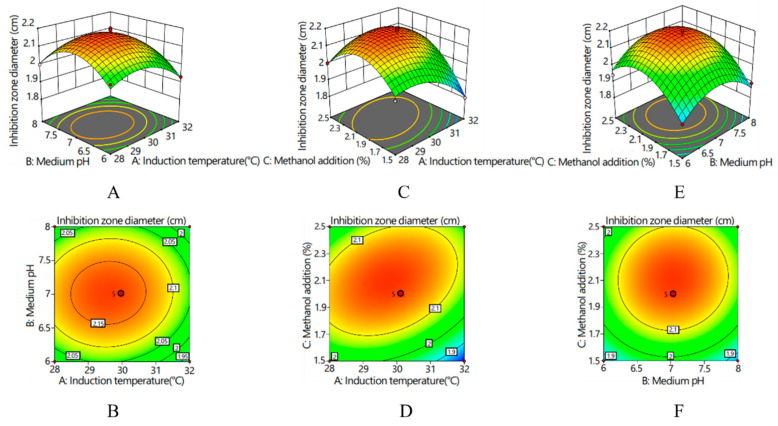
Response surfaces and corresponding contour lines for inhibition zone diameter: (**A**) response surface of the interaction effect between the induction temperature and medium pH on inhibition zone diameter; (**B**) contour line of the interaction effect between the induction temperature and medium pH on inhibition zone diameter; (**C**) response surface of the interaction effect between the induction temperature and methanol addition on inhibition zone diameter; (**D**) contour line of the interaction effect between the induction temperature and methanol addition on inhibition zone diameter; (**E**) response surface of the interaction effect between the medium pH and methanol addition on inhibition zone diameter; and (**F**) contour line of the interaction effect between the medium pH and methanol addition on inhibition zone diameter.

Response surfaces and corresponding contour lines revealed the influences of various factors on the inhibition zone diameter and the interactions between various factors (Figure 4). With an increase in various factors, the inhibition zone diameter presented an increasing and then decreasing trend. The interaction between medium pH and induction temperature was hardly existent (*p* > 0.05); the contour graph was elliptical, and its axes were almost parallel to the coordinate axes (Figure 4A,B). There was a significant interaction between the induction temperature and the amount of methanol added (*p* < 0.05), and the contour graph was elliptical. According to the density of the contour lines, the effect of induction temperature on the inhibitory zone diameter was greater than that of the amount of methanol added (Figure 4C,D). There was no significant interaction between methanol supplemental level and the pH of the medium (*p* > 0.05), and the contour graph was circular (Figure 4E,F). According to the density of the contour lines, the influence of pH of the medium on the diameter of the antibacterial zone was greater than that of methanol addition.

### 2.5. Stability Determination of MaltAtt-1

**Figure 5:**
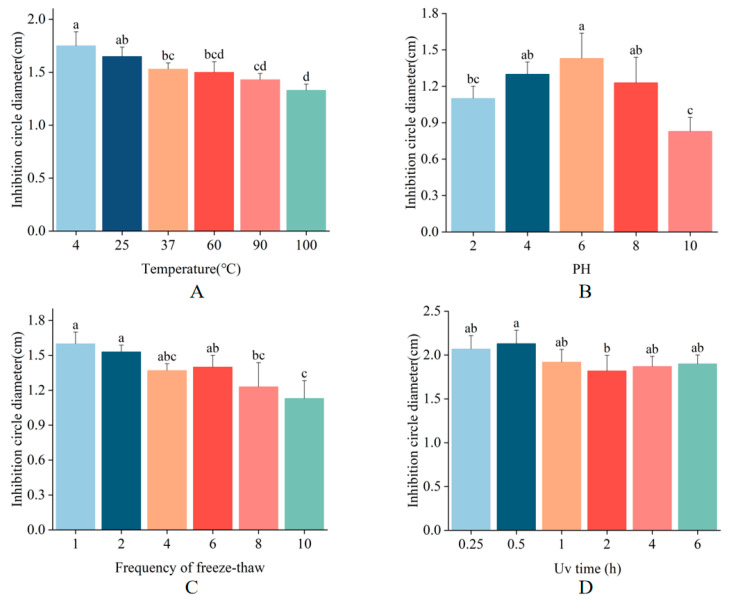
Stability determination of MaltAtt-1: (**A**) thermal stability; (**B**) pH stability; (**C**) freeze–thaw stability; and (**D**) UV stability. Different letters (a–d) above bars denote values that are significantly different from each other (*p* < 0.05, Tukey’s test).

To assess the stability of MaltAtt-1, we evaluated its antimicrobial activity against *E*. *coli* under various conditions, including different temperatures, pH values, UV irradiation durations, and freeze-thaw cycles. MaltAtt-1 demonstrated robust stability at temperatures of 4 °C, 25 °C, and 37 °C. Notably, exposure to high temperatures (100 °C for 15 min) resulted in the inhibition zone diameter being 76% of its maximum, indicating substantial but retained activity (Figure 5A). Regarding pH stability, MaltAtt-1 exhibited strong activity at neutral pH levels but showed reduced effectiveness at extreme pH values (Figure 5B). Antimicrobial peptides are most effective when in specific structural states, with pH significantly influencing their stability [23]. Extreme acidic conditions can alter peptide structure, thereby impacting its efficacy. Upon repeated freeze-thaw cycles, the antimicrobial activity of MaltAtt-1 gradually decreased (Figure 5C). Structural changes during the freezing and thawing processes can lead to the loss of the peptide’s original activity [24]. Furthermore, UV irradiation at varying durations had minimal impact on the stability of MaltAtt-1 (Figure 5D).

### 2.6. Nematocidal Activity Determination of MaltAtt-1

**Figure 6:**
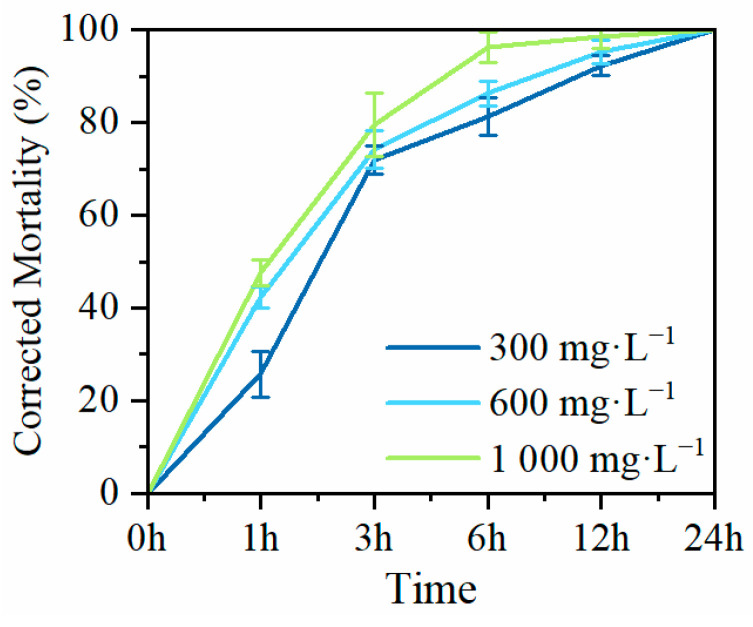
Mortality of MaltAtt-1 against *B*. *xylophilus*.

To investigate the nematocidal activity of MaltAtt-1, produced by secretion expression in *K*. *phaffii* GS115, against PWN (*B*. *xylophilus*), adult nematodes were exposed to purified MaltAtt-1 at different concentrations to determine corrected mortality rates. Exposure to 300 mg·L^−1^ of MaltAtt-1 resulted in a corrected mortality of over 70% (71.94%) within 3 h. At a concentration of 1000 mg·L^−1^, corrected mortality exceed 90% (96.33%) within 6 h. After 24 h of treatment with MaltAtt-1, all nematodes were deceased regardless of the concentration used (Figure 6).

## 3. Discussion

In this study, it was observed that the mutant phenotype host GS115 of *K*. *phaffii* was more suitable for expressing soluble MaltAtt-1 compared to the KM71 host [18,25,26]. The recombinant plasmid pPIC9K-MaltAtt-1 was chemically transformed into *K*. *phaffii* GS115, and induced expression was carried out. SDS-PAGE analysis of the fermentation supernatant confirmed that MaltAtt-1 was successfully secreted into the medium upon induction. Bioinformatics analysis of the antimicrobial peptide MaltAtt-1 indicated that it is a hydrophilic and stable protein, with a significant proportion of β-folds and random coils. After purification through secretion expression in *K*. *phaffii* GS115, MaltAtt-1 exhibited a comparable toxicity effect on *B*. *xylophilus* when compared with the same peptide produced intracellularly by *K*. *phaffii* KM71 [18]. These findings suggest that *K*. *phaffii* GS115 is an effective host for producing soluble MaltAtt-1, which retains its bioactivity against *B*. *xylophilus*, showcasing its potential utility in biotechnological applications involving antimicrobial peptides.

To enhance the expression of recombinant proteins, optimizing induction conditions is crucial. The single-factor method is commonly employed initially due to its simplicity and straightforwardness. However, the interactions of various conditions during the induction of expression are complicated, making it necessary to establish better methods to improve the yield of the target product [27]. Response surface analysis is often conducted after single-factor tests, in order to obtain their results as the foundation for a better response surface method [28]. Through analysis of the functional response surface and contour lines, the optimal process parameters can be determined, and the multi-variable optimization problem can be solved. In this paper, the significance factors affecting the synthetic yield of antimicrobial peptides were selected through a single-factor experiment and optimized by a central combination experiment. The regression equation was obtained, as well as the optimal solution. The results of the response surface experiment were verified, and the average value of the diameter of the inhibitory zone was 2.10 cm, similar to the result predicted by the model, indicating that the obtained regression model can better predict the influence of the inhibitory zone diameter. The results showed that the response surface method could optimize the expression conditions for recombinant yeast, thus increasing the expression of target polypeptides.

In the process of production and application, antimicrobial peptides can be affected by a variety of environmental factors, such as temperature and pH [29,30]; therefore, this study focused on the stability of MaltAtt-1 against such factors above to determine preferable culture conditions. MaltAtt-1 adapted to high temperatures and UV irradiation without losing antimicrobial activity, indicating that these antimicrobial peptides have strong thermal and irradiation stability. Studies have shown that electric charge is an important factor in determining the bioactivity of antimicrobial peptides, as bacterial cell membranes are generally negatively charged, while antimicrobial peptides are positively charged [31]. When the environmental pH value changes, the distribution of electric charge will be altered, causing variations in the secondary and tertiary structure of the antimicrobial peptide, resulting in different kinds of interactions between the antimicrobial peptide and bacterial cell membrane, affecting the antibacterial efficacy [32,33]. MaltAtt-1 had good stability at neutral pH levels and could not maintain good activity at inappropriate pH. During the freeze-thaw process, the abnormal configuration of peptide chains may occur, causing the form with functional potency to be gradually destroyed, along with a decline in antimicrobial activity [24], which was observed in this study.

A sound production method and suitable storage conditions are necessary for the production and application of new nematicides. This study established a new eukaryotic secretion expression system for MaltAtt-1 and investigated its stability. The environmental variable range that can ensure its high antibacterial activity was clarified, providing a sufficient theoretical basis for the synthesis and preservation of MaltAtt-1, which is beneficial for its subsequent production and application, ultimately providing a new approach for the biological control of pine wilt disease.

## 4. Materials and Methods

### 4.1. Strain and Plasmid

The recombinant plasmid pPIC9K-MaltAtt-1 used in this study was constructed in a previous study [18]. *K*. *phaffii*, strain GS115 (Angyubio, Shanghai, China), was used for the expression of MaltAtt-1.

### 4.2. Transformation and Expression of Recombinant Protein MaltAtt-1 in K. phaffii

The recombinant plasmids were linearized with *Sac* I enzyme (Takara, Beijing, China) and chemically converted into *K*. *phaffii* GS115. The positive yeast transformants of MaltAtt-1 were cultivated in yeast extract peptone dextrose (YPD) medium at 30 °C overnight. Then, 5% of this culture was inoculated into 50 mL buffered glycerol-complex (BMGY) medium at 200 rpm and 30 °C for 24 h until the OD_600_ was close to 3.0. Cells were harvested by centrifugation at 5000 rpm for 10 min at room temperature and then resuspended in buffered methanol-complex medium (BMMY) to induce expression of the recombinant proteins. The resuspended culture was grown for 48 h with the addition of 1% methanol (Xilong, Guangzhou, China) every 24 h, followed by centrifugation at 6500 rpm for 10 min. The fermentation supernatant was collected to identify the expression of recombinant MaltAtt-1 through sodium dodecyl sulfate–polyacrylamide gel electrophoresis (SDS-PAGE) according to the steps detailed in Section 4.5. A supernatant of *K*. *phaffii* GS115 with an empty vector used as a control. Then, the antimicrobial activity of the fermentation supernatant was determined according to the steps detailed in Section 4.6.

### 4.3. Bioinformatics Analysis of Antimicrobial Peptide MaltAtt-1

The physicochemical properties and hydrophobicity of MaltAtt-1 were predicted by Expasy (https://www.expasy.org/, accessed on 14 March 2024), a website for the bioinformatics analysis of antimicrobial peptides. The secondary structure was predicted using SOPMA (https://npsa-pbil.ibcp.fr/cgi-bin/npsa_automat.pl?page=npsa_sopma.html, accessed on 14 March 2024), and the SWISS-MODEL online program (https://swissmodel.expasy.org/interactive/, accessed on 14 March 2024) was used to to predict the tertiary structure of the antibacterial peptide MaltAtt-1.

### 4.4. Optimization of Recombinant Protein MaltAtt-1 in K. phaffii

In order to achieve a high level of production of the recombinant protein, shake bottle induction conditions were optimized to explore the expression levels of recombinant protein under different induction times (24 h, 48 h, 72 h, 96 h, and 120 h), induction temperatures (24 °C, 26 °C, 28 °C, 30 °C, and 32 °C), initial pH values (4.0, 5.0, 6.0, 7.0, and 8.0), methanol volume fractions (0.5%, 1.0%, 1.5%, 2.0%, and 2.5% *v*/*v*), and initial glycerol concentrations (0.5%, 1%, 3%, 5%, and 7% *v*/*v*). After induction, the supernatant was collected to explore the influence of various factors on the expression level of the recombinant protein. The protein concentration and inhibition circle diameter were used as indicators. Each experiment were performed in triplicate.

### 4.5. Polyacrylamide Gel Electrophoresis Analysis

Polyacrylamide gel electrophoresis was conducted to verify the expression of the antimicrobial peptide. FuturePAGE^TM^ prefabricate protein glue (ACE Biotechnology, Changzhou, China) was placed in an electrophoresis tank according to the standard operation process, the internal and external tanks were filled with 1* protein electrophoresis buffer (ACE Biotechnology, Changzhou, China), and the preparation of samples was as follows. The protein sample was mixed with the sample buffer (MeilunBio, Dalian, China), then boiled in a boiling water bath for 10 min and centrifuged at room temperature at 12,000 rpm for 10 min. The samples were taken in sequence, and the electrophoresis apparatus was started with the appropriate voltage and time selected for electrophoresis. After electrophoresis, Coomassie bright blue staining was performed to measure the concentration of the protein and, finally, the gel image was used for imaging.

### 4.6. Antimicrobial Activity Detection of Recombinant Protein MaltAtt-1

The agar cavity diffusion method was used to verify the antimicrobial activities of MaltAtt-1 [34,35]. A total of 100 μL of *E*. *coli* fluid was spread on an LB solid medium with a diameter of 9 cm; a hole with a diameter of 1 cm were drilled in the medium, followed by adding 100 μL of the supernatant of *K*. *phaffii* GS115 with the recombinant vector. The medium was cultured at 37 °C for 12 h. *E. coli* was stored in the Key Laboratory of Integrated Pest Management in Ecological Forests, Fujian Agriculture and Forestry University. ddH_2_O was used as the empty control, and the supernatant of *K*. *phaffii* GS115 with an empty vector was used as the negative control.

### 4.7. Experimental Design by Response Surface Methodology

Based on the results of the single-factor test, pH, induction temperature, and methanol volume fraction were determined to be the main influencing factors, with the inhibition zone diameter as the index. Based on the Box–Behnken (BBD) central combination design principle, these factors were selected as independent variables, and the inhibition zone diameter was set as the response value. An orthogonal experiment with a three-factor, three-level design was set up. The data obtained were processed using the Design-Expert 8.0 software, in order to build a response surface model. The experimental factors and encoding levels of response surface experiments are detailed in Table 3.

### 4.8. Stability Determination of Recombinant Protein MaltAtt-1

#### 4.8.1. Thermal Stability Assay

To investigate the thermal stability of the recombinant protein fermentation supernatant, an antibacterial activity test was performed. The fermentation supernatant was treated in a water bath at 4 °C, 25 °C, 37 °C, 60 °C, 90 °C, or 100 °C for 15 min, then placed in an ice bath for rapid cooling. Then, antimicrobial activity was tested to assess the stability at various temperatures. Fermentation supernatant stored at room temperature was used as the control. The experiments were performed in triplicate.

#### 4.8.2. pH Stability

The fermentation supernatant was mixed with PBS buffer (Coolaber, Beijing, China) at pH 2.0, 4.0, 6.0, 8.0, or 10.0 in an equal proportion for 1 h, and then NaOH (Xilong, Guangzhou, China) or HCl (Xilong, Guangzhou, China) solutions were used to adjust the pH to 7.0 in the case of bacteria. After the treatment, antimicrobial activity was tested to assess the stability at various pH. The supernatant mixed with PBS at pH 7.0 was used as the control. Each experiment was performed in triplicate.

#### 4.8.3. Freeze-Thaw Stability

To investigate the freeze-thaw stability of recombinant protein fermentation supernatant, an antibacterial activity test was performed. The fermentation broth was repeatedly frozen and thawed at −20 °C either 1, 2, 4, 6, 8, or 10 times. Then, its activity was determined through an antibacterial zone test. Untreated antimicrobial peptides were used as the control group. Each experiment was performed in triplicate.

#### 4.8.4. UV Stability

To investigate the UV stability of the recombinant protein fermentation supernatant, an antibacterial activity test was performed. The fermentation supernatant was subjected to ultraviolet irradiation for 15 min, 30 min, 1 h, 2 h, 4 h, or 6 h. Then, its activity was determined through an antibacterial zone test. Untreated antimicrobial peptides were used as the control group. Each experiment was performed in triplicate.

### 4.9. Nematocidal Activity Assay of MaltAtt-1

Nematocidal activity assay of MaltAtt-1 was performed according to Chu et al. [18]. A Coolaber^®^ His tag protein purification kit (Beijing, China) was used to purify MaltAtt-1 from supernatant following the recommended steps. Purified MaltAtt-1 and 10 μL of *B*. *xylophilus* suspension included 100 adults were added into a 24-well culture plate, and sterile water was used to dilute the antimicrobial peptide until its concentration reached 300 mg·L^−1^, 600 mg·L^−1^, or 1000 mg·L^−1^. Sterile water was used as a control. The culture plate was incubated at 26 °C, and after 3 h, 6 h, 12 h, and 24 h, the number of dead nematodes was counted under the microscope. Mortality was calculated by dividing the death number by the original amount and was corrected using Abbott’s formula. *B*. *xylophilus* adults were raised from strain Nx11 in the Key Laboratory of Integrated Pest Management in Ecological Forests, Fujian Agriculture and Forestry University, Fuzhou, China.

## 5. Conclusions

In this study, the soluble expression of MaltAtt-1 from *K*. *phaffii* stain GS115 was realized with a high toxicity effect on *B*. *xylophilus*. The optimal conditions for MaltAtt-1 induced expression were determined through a central combination experiment, which were an induction time of 72 h, an induction temperature of 30 °C, a culture medium of pH 7.6, a methanol volume fraction of 2.0%, and an initial glycerol concentration of 1%, respectively. MaltAtt-1 showed good stability at a wide range of temperatures, various UV intensities, and in a neutral pH environment, while repeated freeze–thawing significantly decreased the stability of MaltAtt-1.

## Figures and Tables

**Table 1:** Results of physicochemical properties analysis of the antimicrobial peptide MaltAtt-1.

Index	Argument
Amino acid quantity	113
Molecular formula	C_528_H_782_N_164_O_158_
Relative molecular mass	11,955.02
Theoretical isoelectric point	9.69
Asp + Glu	7
Arg + Lys	11
Coefficient of instability	13.68
Aliphatic coefficient	50.97
GRAVY	−0.595

**Table 2:** Response surface variance analysis.

Source	Sum of Squares	df	Mean Square	F Value	Prob > F	Significance
Model	0.2281	9	0.0253	23.33	0.0002	**
A	0.0091	1	0.0091	8.39	0.0231	*
B	0.0003	1	0.0003	0.2876	0.6084	
C	0.0288	1	0.0288	26.51	0.0013	**
AB	0.0002	1	0.0002	0.2071	0.6628	
AC	0.0144	1	0.0144	13.25	0.0083	**
BC	0.0004	1	0.0004	0.3682	0.5632	
A^2^	0.0285	1	0.0285	26.22	0.0014	**
B^2^	0.0579	1	0.0579	53.28	0.0002	**
C^2^	0.0709	1	0.0709	65.25	<0.0001	**
Residual	0.0076	7	0.0011			
Lack of fit	0.0039	3	0.0013	1.42	0.3601	
Pure error	0.0037	4	0.0009			
Cor total	0.2358	16				
R^2^ = 0.9677; R^2^_Adj_ = 0.9263						

Note: * Significant difference (*p* < 0.05); ** extremely significant difference (*p* < 0.01).

**Table 3:** Influencing factors and their encoding in response surface experiments.

Influencing Factors	Encoding Level		
	−1	0	1
(A) induction temperature/°C	28	30	32
(B) medium pH	6.0	7.0	8.0
(C) methanol addition/%	1.5	2.0	2.5

## Data Availability

Data is contained within the article and supplementary material.

## Supplementary Materials

The following supporting information can be downloaded at: https://www.mdpi.com/article/10.3390/ijms25168555/s1.
