# Supplementary material for: Optimization and Stability Assessment of Monochamus alternatus Antimicrobial Peptide MaltAtt-1 in Komagataella phaffii GS115 for the Control of Pine Wood Nematode
Source: Int J Mol Sci. 2024 Aug 6;25(16):8555. doi: 10.3390/ijms25168555 (PMC11354690; doi:10.3390/ijms25168555)
Supplement: Supplementary file 1 [file ijms-25-08555-s001.zip › ijms-3051375-supplementary.pdf]

Table S1. Orthogonal experiment results.

number	A induction temperature	B medium pH	C methanol addition	Y inhibition zone diameter (cm)
1	1 (32)	0 (7.0)	-1 (1.5)	1.80
2	0 (30)	-1 (6.0)	1 (2.5)	1.94
3	1 (32)	0 (7.0)	1 (2.5)	2.07
4	0 (30)	0 (7.0)	0 (2.0)	2.19
5	-1 (28)	0 (7.0)	1 (2.5)	2.00
6	0 (30)	0 (7.0)	0 (2.0)	2.20
7	0 (30)	1 (8.0)	-1 (1.5)	1.89
8	1 (32)	-1 (6.0)	0 (2.0)	1.93
9	0 (30)	0 (7.0)	0 (2.0)	2.19
10	0 (30)	0 (7.0)	0 (2.0)	2.13
11	-1 (28)	-1 (6.0)	0 (2.0)	2.03
12	1 (32)	1 (8.0)	0 (2.0)	1.93
13	0 (30)	-1 (6.0)	-1 (1.5)	1.87
14	-1 (28)	0 (7.0)	-1 (1.5)	1.97
15	-1 (28)	1 (8.0)	0 (2.0)	2.00
16	0 (30)	1 (8.0)	1 (2.5)	2.00
17	0 (30)	0 (7.0)	0 (2.0)	2.15
